# Mixed 3D–2D Perovskite Flexible Films for the Direct Detection of 5 MeV Protons

**DOI:** 10.1002/advs.202204815

**Published:** 2022-11-27

**Authors:** Laura Basiricò, Ilaria Fratelli, Matteo Verdi, Andrea Ciavatti, Luisa Barba, Olivia Cesarini, Giorgio Bais, Maurizio Polentarutti, Massimo Chiari, Beatrice Fraboni

**Affiliations:** ^1^ Department of Physics and Astronomy University of Bologna Bologna 40127 Italy; ^2^ National Institute for Nuclear Physics INFN section of Bologna Bologna 40127 Italy; ^3^ National Council of Research Institute of Crystallography Trieste 34149 Italy; ^4^ National Institute for Nuclear Physics INFN Laboratori Nazionali di Legnaro Legnaro 35020 Italy; ^5^ Elettra‐Sincrotrone Trieste Trieste 34149 Italy; ^6^ National Institute for Nuclear Physics INFN section of Firenze Sesto Fiorentino 50019 Italy

**Keywords:** 2D‐3D perovskites, dosimetry, flexible radiation detectors, perovskites, proton detectors, thin films

## Abstract

This study reports on a novel, flexible, proton beam detector based on mixed 3D–2D perovskite films deposited by solution onto thin plastic foils. The 3D–2D mixture allows to obtain micrometer‐thick and highly uniform films that constitute the detector's active layer. The devices demonstrate excellent flexibility with stable electric transport properties down to a bending radius of 3.1 mm. The detector is characterized under a 5 MeV proton beam with fluxes in the range [4.5 × 10^5^ – 1.4 × 10^9^] H^+^ cm^−2^ s^−1^, exhibiting a stable response to repetitive irradiation cycles with sensitivity up to (290 ± 40) nC Gy^−1^mm^−3^ and a limit of detection down to (72±2) µGy s^−1^. The detector radiation tolerance is also assessed up to a total of 1.7 × 10^12^ protons impinging on the beam spot area, with a maximum variation of the detector's response of 14%.

## Graphical abstract

The direct detection of 5 MeV protons by flexible devices based on 3D–2D mixed perovskite is reported here. The mixture allows to directly deposit onto polymeric foils micrometer‐thick and highly uniform films that constitute the detector's active layer. This sensor can detect the dose of absorbed protons producing an electrical output signal.

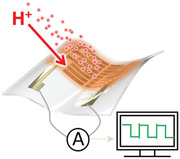

## 1 Introduction

The monitoring of proton beam's flux, energy and position is of utmost importance in various fields, such as beam control during fundamental physics experiment and for personal dosimetry during hadron therapy treatments, the cutting‐edge medical tool for cancer therapy.^[^ ^1^ ^]^ Silicon based devices, e.g. MOSFETs, demonstrated their reliability as real‐time dosimeters, but their scaling up to cover useful areas requires complex and expensive fabrication procedures.^[^ ^2^ ^]^ Plastic scintillators and scintillating fibres are also used for proton beam diagnostics and real‐time dosimetry, but they need to be coupled to photomultiplier tubes or silicon photodiodes with a suitable readout chain to enhance the light sensitivity and obtain a reliable detection.^[^ ^3^, ^4^ ^]^ Moreover, complex calibration procedures are generally required and sometimes the simultaneous detection with different scintillators is needed for achieving accurate measurements. Finally, mechanical flexibility is a relevant requirement unsolved by detectors currently available on the market.

Research on innovative materials for the detection of ionizing radiation has rapidly grown in the last ten years, focusing on the classes of materials that allow overcoming the main constraints of traditional detectors, i.e., their mechanical stiffness and difficulty to implement them into large‐area pixelated detector matrixes at limited costs. Organic semiconductors and perovskites share the property of being processable from solution by low‐cost and low‐temperature deposition techniques, addressing the challenge of covering large areas at affordable costs on thin flexible plastic substrates. Both these materials have recently demonstrated excellent direct detection performances for high energy photons^[^ ^5^, ^6^ ^]^ and alpha particles^[^ ^7^, ^8^, ^9^ ^]^, and more recently for fast and thermal neutrons.^[^ ^10^, ^11^, ^12^ ^]^ The direct proton beam detection or dose‐monitoring by perovskite based devices has not been explored yet and only one paper has been published so far on direct proton detection, implemented by fully organic flexible devices.^[^ ^13^ ^]^

In this work, we propose a novel flexible proton detector based on mixed 3D and 2D perovskites films deposited from solution. Mixed 3D–2D perovskites are formed by mixing 3D (based on methylammonium (MA) cations) and 2D (based on larger organic ammonium (OA) cations) structure perovskites. Their employment has been reported as an effective strategy to retain the exceptional transport properties of 3D perovskites and the high stability induced by the layered structure of 2D perovskites.^[^ ^14^ ^]^ We recently demonstrated how 2D (PEA)_2_PbBr_4_ (PEA = C_6_H_5_C_2_H_4_NH_3_^+^) perovskite films can be employed as active layer for flexible X‐ray direct detectors with high performance.^[^ ^15^ ^]^ By adding the 3D phase (MAPbBr_3_) we here aim to enhance the radiation absorption of protons by the perovskite film, thanks to the higher density of MAPbBr_3_ and to the higher thickness of the active layer for mixed compounds.

The here proposed devices demonstrate an accurate monitoring of proton dose with instant feedback and low limit of detection, and provide a stable response even after hard and long‐lasting proton irradiation. They also show stable transport properties under bending and fatigue tests down to curvature radius of 3.1 mm. The presented results provide an effective solution to the challenge of identifying novel functional materials and portable devices for real‐time accurate monitoring of proton dose, addressing the quest for low‐cost scalability over large areas and mechanical flexibility, still unsolved for a range of application which span from personal dosimetry to large area and lightweight detectors for large accelerators facilities and space missions.

## 2 Results

### 2.1 Mixed 3D–2D Perovskite Flexible Detectors

**Figure 1:**
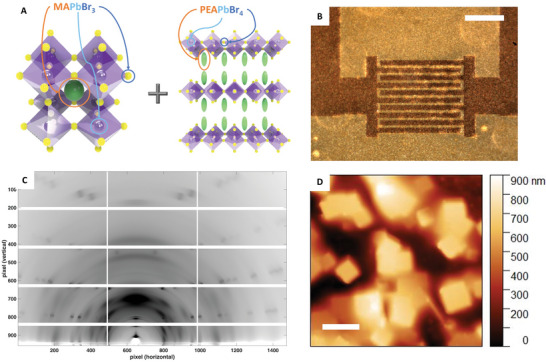
Mixed 3D–2D perovskite film active layer on PET flexible substrate: structure and morphology. A) Schematics of MAPbBr_3_ (3D) and (PEA)_2_PbBr_4_ (2D) perovskite composing the 3D–2D perovskite film under study. B) Optical microscope image of the detector's active layer. Scale bar: 500 µm. C) GI‐XRD data of 3D/2D perovskite film composed by MAPbBr_3_ and (PEA)_2_PbBr_4_ at 2D:3D 35% precursors volume ratio. D) AFM image of the mixed 3D–2D perovskite film at 2D:3D 35% precursors volume ratio. Scale bar: 5 µm.

The mixed 3D–2D perovskite film‐based proton detectors have been fabricated by processing the perovskite mixture from solution as detailed in Experimental Method section, and then depositing the perovskite layer by spin coating onto a flexible 125 µm poly(ethylene terephthalate) (PET) foil with prepatterned Cr/Au interdigitated electrodes by lithographic techniques (see Experimental section for detail). As known from literature, mixed 3D–2D perovskites have both MAPbBr_3_ (3D) and (PEA)_2_PbBr_4_ (2D) perovskite crystalline structure (**Figure** **1**A)^[^ ^14^, ^16^ ^]^. By tuning the 2D:3D ratio with a 35% of 2D precursor component we were able to obtain a homogeneous coverage of the detector's active area (Figure 1B). To examine the crystal structure of the mixed 3D–2D perovskite film, we performed grazing‐incidence X‐ray diffraction (GI‐XRD) measurements at the X‐ray Diffraction beamline 5.2 at the Synchrotron Radiation Facility Elettra in Trieste (Italy) (Figure 1C). As expected, the pattern shows the characteristic peaks of MAPbBr_3_ and (PEA)_2_PbBr_4_ as well as those of the PET substrate. However, due to the presence of (PEA)_2_PbBr_4_, the perovskite films show an ordered layer structure in out‐of‐plane direction, with poor long‐range order in the a and b directions (Figure S1, Supporting Information), in accordance with literature.^[^ ^16^ ^]^ The top morphology of the film, investigated through AFM measurements and reported in Figure 1D, clearly shows the cubic microcrystals peculiar of the MAPbBr_3_ component (about 2 µm lateral dimensions), which spontaneously crystallize within the film. The MAPbBr_3_ microcrystals are embedded in a continuous layer of (PEA)_2_PbBr_4_. Thanks to the presence of (PEA)_2_PbBr_4_ phase, mixed 3D–2D perovskite films result about twofold thicker than films with MAPbBr_3_ microcrystals only, i.e., an average thickness of 4 µm is estimated from AFM profile (Figure S2, Supporting Information), confirming the uniform coverage of the film with no pinholes, as also assessed by optical microscopy.

**Figure 2:**
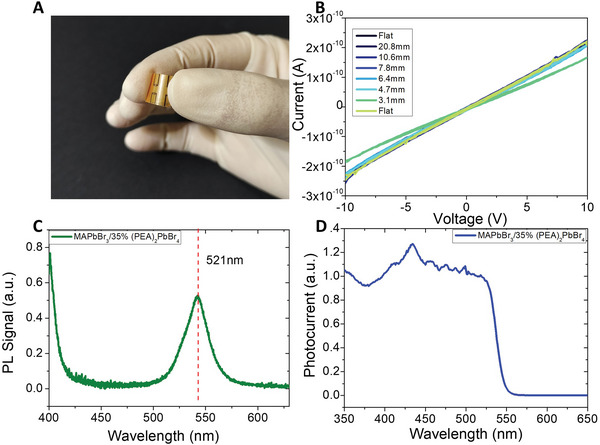
Electrical and optoelectronic properties of the 3D–2D perovskite active layer. A) Image showing the flexibility of the device. B) Plot of the current versus voltage measurements of the detector in pristine flat condition, under bending down to 3.1 mm radius, and then kept again in flat condition. Details on the experimental setup employed are shown in Figure S3B,C (Supporting Information). C) PL spectrum of 3D–2D perovskite films on PET substrate. The peak at *λ* ≈ 400 nm is not entirely visible due to a cutoff filter needed to remove the LASER reflection from the spectrum during acquisition. D) UV–vis photocurrent spectrum of the mixed 3D–2D perovskite films on PET substrate.

The flexibility of the fabricated devices is shown in **Figure** **2**A. The electrical characteristics of the mixed 3D–2D perovskite film tested under bending exhibited excellent stability down to a bending radius of 4.7 mm, compatible to human body curves in view of possible medical personal dosimetry application. At bending radius of 3.1 mm the current starts to degrade. However, the detector completely recovers its current pristine value once kept again in flat condition (Figure 2B). The device also demonstrates outstanding stability to the mechanical stress provided by multiple bending cycles, with unvaried current versus voltage curves after up to 50 bending cycles at 4.7 mm radius, as shown in Figure S3A (Supporting Information). Such excellent stability of the electric characteristics under bending indicates that both the transport properties of the perovskite active layer and the ohmic nature of the electric contact at the interface between the perovskite and injecting/collecting electrodes are not affected by such a mechanical stress, proving the reliability of the here reported device as flexible detector.

The optoelectronic properties of the mixed 3D–2D perovskite film were investigated to further evaluate its suitability for ionizing radiation detection. The photoluminescence (PL) spectrum of perovskite films (Figure 2C) exhibits the characteristic peak of MAPbBr_3_ microcrystals^[^ ^17^ ^]^ at *λ* ≈ 521 nm and that of (PEA)_2_PbBr_4_ at *λ* ≈ 400 nm,^[^ ^18^ ^]^ assessing the co‐existence of the two phases in the compound. Due to the presence of both MAPbB_3_ and (PEA)_2_PbBr_4_ phases, experimentally assessed by structural (GI‐XRD measurements) and optoelectronic (PL spectra) analyses, we calculated its electron‐hole pair creation mean energy (*W*), as the weighted average of the pair creation energies of the two compounds, i.e., as $W_{\mathrm{mixed}}=\left(W_{{\mathrm{MAPbBr}}_{3}}+0.35W_{{\left(\mathrm{PEA}\right)}_{2}{\mathrm{PbBr}}_{4}}\right)/1.35$, obtaining *W*_mixed_ ≈ 6.38 eV. The *W* of each perovskite has been predicted according to the empirical model of Devanathan and co‐authors^[^ ^19^ ^]^

(1) $$W=2E_{G}+1.43\;\mathrm{eV}$$

where *E*_G_ indicates the energy gap of the semiconductor. We extracted from the UV‐Vis photocurrent spectrum the energy gap of the mixed perovskite film *E*_G_ ≈ (2.29 ± 0.08) eV^[^ ^20^ ^]^, and we estimate the electron‐hole pair creation energy, $W_{{\mathrm{MAPbBr}}_{3}}=\left(6.02\pm \;0.16\right)\;\mathrm{eV}$ (Figure 2D and Figure S4a, Supporting Information). These values are in good agreement with the values reported for MAPbBr_3_ single crystals^[^ ^21^ ^]^, assuring that the optoelectronic properties of the 3D perovskite have been preserved in the mixture film. The energy gap value of (PEA)_2_PbBr_4_ of *E*_G_ = (3.00± 0.03) was extracted from our photocurrent spectra (Figure S4b, Supporting Information) and from the literature^[^ ^22^ ^]^, resulting in a $W_{{\left(\mathrm{PEA}\right)}_{2}{\mathrm{PbBr}}_{4}}=\left(7.43\pm 0.06\right)\;\mathrm{eV}$. The theoretical maximum value of sensitivity per unit volume corresponding to the estimated *W*_mixed_ can be calculated as *S*_V_ = *qρ*/*W* ^[^ ^23^ ^]^. For the mixed 3D–2D perovskite under study this value results *S*_Vmixed_ = 533 nC Gy^‐1^mm^‐3^, close to that of Si (637 nC Gy^‐1^mm^‐3^) and much higher than that of diamond (217 nC Gy^‐1^mm^‐3^) the two benchmark materials for on‐line dosimeters. It is noteworthy that, if the presence of 2D‐perovskite lowers the total sensitivity due to its low density (2.27 g cm^−3^)^[^ ^24^ ^]^ and large band‐gap, on the opposite it allows to significantly lower the dark current (see Figure S5, Supporting Information) and to achieve micrometer thick layers, homogeneously covering the detector's active area, a very challenging goal to achieve with a pure MAPbBr_3_ active layer.

### 2.2 Direct Detection of 5 MeV Proton Beam

**Figure 3:**
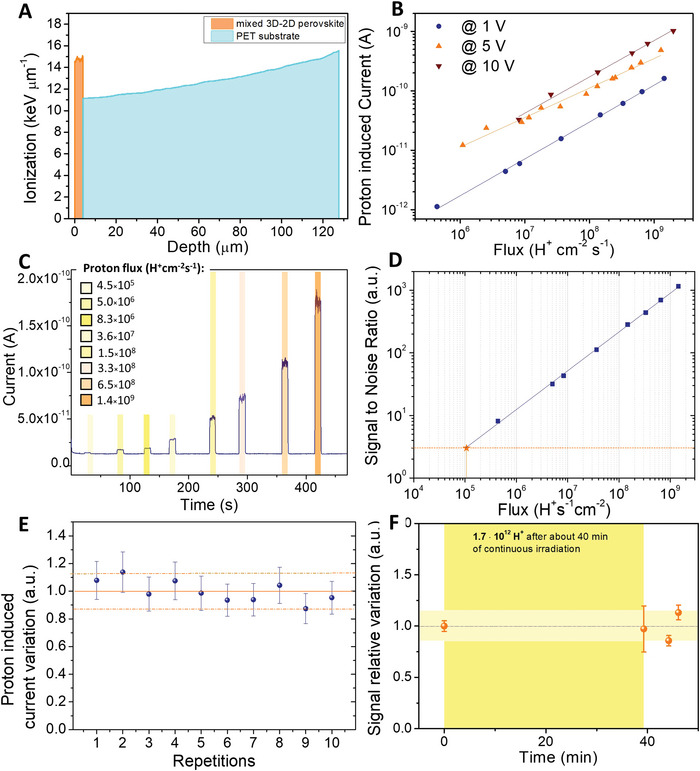
Proton detection by mixed 3D–2D film‐based devices on flexible substrates. A) Simulated curve of the energy released by the proton beam in the detector layers. B) Plot in logarithmic scale of the signal amplitude, i.e., the current induced by 10 s proton irradiation at 1 V (blue circles), 5 V (orange triangles), and 10 V (upside down red triangles). C) Plot of the dynamic current response in function of time for the detector biased at 1 V and irradiated with 10 s proton beam shots. The colored boxes indicate the increasing proton fluxes employed, in the range [4.5 × 10^5^–1.4 × 10^9^] H^+^ cm^−2^ s^−1^. D) Plot of the SNR in function of the proton flux. The blue squares are experimental data, the orange dashed line corresponds to SNR = 3, the orange star indicated the extrapolated LoD value. E) Stability of the proton induced current variation up to 10 repetitions of 10 s irradiation cycles. During the irradiation procedure the device has been biased at 5 V. Each data point has been normalized for the impinging proton flux and for the mean value. The uncertainty associated to each point has been evaluated from the root sum square of the statistic uncertainty of the proton induced, current value, the one associated to the proton flux and the one of the mean value. The dashed orange lines identify the maximum semi‐dispersion around the mean value, i.e., half of the difference between the maximum and the minimum value of the data set. F) Radiation hardness test. The response of the detector biased at 5 V measured in pristine condition and after 40 min of irradiation tests, with a total of 1.7 × 10^12^ protons impinging on the beam spot area. The measurement was then repeated after few minutes.

The response under proton beams of the mixed 3D–2D film‐based detectors was characterized at the LABEC ion beam center (Laboratory of Nuclear Techniques for the Environment and Cultural Heritage, INFN Firenze, Italy), employing a 5 MeV proton beam extracted into atmosphere, provided by the 3 MV Tandetron accelerator.^[^ ^25^ ^]^ The proton flux, in the range of [10^5^ – 10^9^] H^+^ s^−1^ cm^−2^, is tuned by changing the proton beam current between 0.01 pA and 49 pA. A rotating chopper, intercepting the beam between the extraction window and the sample, is used to monitor and measure such low proton current values.^[^ ^26^ ^]^ The experimental setup used during the proton detection tests is reported in Figure S4 (Supporting Information), further details are reported in Materials and Methods section. Before impinging on the sample, the 5 MeV proton beam loses about 390 keV, as calculated through the stopping and range of ions in matter (SRIM) Monte Carlo code^[^ ^27^ ^]^, by passing through several layers between the beam exit point and the perovskite detector, such as 200 nm of Si_3_N_4_ for the beam extraction window, 8 mm of mixed air‐He (50–50%) atmosphere in the gap between the extraction window and the metal box containing the sample, 14 µm of Al foil for the entrance window of the box, and 14 mm of air inside the box. The linear energy transfer (LET) released by each proton inside the 4 µm thick mixed 3D–2D perovskite layer and inside the 125 µm PET substrate has been calculated by SRIM simulation, resulting about of 59 keV and 1550 keV respectively, as shown in the graph of LET in function of the penetration depth reported in **Figure** **3**A. Correspondent range plot is reported in Figure S7 (Supporting Information). The detector's response to the proton beam was characterized by measuring the current flowing between the electrodes upon 10 s irradiation cycles at different bias voltages, i.e., 10, 5, and 1 V, all compatible with battery operation, allowing to envisage wearable personal dosimetry application. The proton induced current is proportional to the impinging proton flux for all the bias voltages tested (Figure 3B and Figure S8, Supporting Information). Figure 3C reports the current flowing in the detector upon 10 s irradiation shots of increasing fluxes between 4.5 × 10^5^ H^+^ cm^−2^ s^−1^ and 1.4 × 10^9^ H^+^ cm^−2^ s^−1^. Even at a bias as low as 1 V, the detector's response to protons is fast and box‐shaped within the wide range of proton fluxes employed for the characterization. The experimental sensitivity per unit volume, calculated as the first derivative of the proton induced current (*I*) versus dose rate (*Dr*) $\left(S_{V}=\frac{dI}{dDr}\;\frac{1}{V}\right)$, resulted up to (290 ± 40) nC Gy^−1^mm^−3^. We attribute such a value, lower than the theoretical one, to an incomplete collection of the charges produced in the film, possibly due to trapping effects in the device's channel. This result however confirms that the charge collection of electron‐hole pairs created by ionization constitutes the most significant contribution to the current signal recorded under proton irradiation. It is worth noting that the characteristic proton beam aperture time at LABEC, of about 100 ms, limits a precise evaluation of the response time of the here studied mixed 3D–2D perovskite detector, which clearly follows the timing of the beam aperture and of the rotating chopper employed for the proton beam current estimation (Figure S9, Supporting Information). However, with the aim of providing an indication for the potential time response of the detector, we measured the photoresponse of the detector to a 375 nm LASER illumination (Figure S10, Supporting Information). The rise (fall) time, that we considered as the time interval needed to increase the photocurrent from 10% to 90% of its maximum value (decrease from 90% to 10%), resulted of 32 µs (275 µs). Such values are in line with typical values reported for MAPbBr_3_ single crystals.^[^ ^28^, ^29^ ^]^

After each proton irradiation cycle, the current completely recovers to its pristine values. It is noteworthy that, in our previous work on flexible organic thin film‐based direct proton detectors fabricated onto PET foils^[^ ^13^ ^]^, we observed a different behaviour, i.e., when the beam was switched off, the current had an initial fast drop due to the recombination of the charges, followed by a slower decay. This behaviour led to a gradual shift of the baseline scaling with the total dose received by the detector. We attributed such behaviour to the accumulation of proton‐induced charges in the plastic substrate, which, acting as a virtual bottom‐gate for the organic semiconductor layer, increased its electrical conductivity. We did not observe such an effect in the here reported mixed 3D–2D perovskite detectors, fabricated on plastic substrate of the same thickness, suggesting that the electrically active states responsible of the persistent current observed for organic semiconductors active layer are here totally absent. Therefore, the here proposed mixed 3D–2D perovskite detector can monitor the impinging proton flux in real‐time with a complete recovery of its pristine conditions after few hundreds of milliseconds from a previous irradiation.

The minimum detectable dose for a radiation detector is generally expressed through the LoD (Limit of Detection) parameter, defined as the minimum intensity of radiation which provides a signal­ to ­noise ratio (SNR) equal to 3, following the IUPAC standard.^[^ ^30^ ^]^ To estimate the LoD, the SNR was plotted as function of proton flux in Figure 3D and the LoD value was then linearly extrapolated from the plot. For 1 V bias the LoD resulted as low as (1.06±0.03) × 10^5^ H^+^s^−1^cm^−2^, a value near the actual minimum flux measured. From the extrapolation of data collected at higher bias, much lower LoD values resulted, i.e., (3.0±0.4) × 10^3^ H^+^s^−1^cm^−2^ and (6.0±0.6) × 10^3^ H^+^s^−1^cm^−2^ for 5 V and 10 V applied bias voltage respectively (Figure S11, Supporting Information). However, such values are further away than the minimum data experimentally recorded and therefore less accurate.

The dose rate corresponding to each proton flux can be estimated through the equation:

(2) $$Dr=\frac{E_{\mathrm{abs}}N_{p}}{\rho_{\mathrm{mixed}}V}$$

where *E*_abs_ is the energy absorbed by the active layer calculated with SRIM Monte Carlo simulations, i.e., 59 keV released in 4 µm thick mixed 3D–2D perovskite layer; *N_p_* is the proton flux; *ρ*_mixed_ is the density of the mixed 3D–2D perovskite, calculated as the weighted average between the 3D and the 2D perovskite composing the mixture and resulting *ρ*_mixed_ = 3.43 g cm^−3^, and *V* is the volume of the active layer, considered as the product of the perovskite layer thickness (4 µm) and the whole area of the detector (1 mm^2^), which is entirely contained within the beam spot (17 mm^2^). The as converted minimum detectable dose rate resulted (729.2±0.2) µGy s^−1^ at 1 V, and (72±3) µGy s^−1^ and (144±4) µGy s^−1^ at 5 V and 10 V respectively. These values are from 1 to 2 orders of magnitude lower than the ones we reported for organic film based proton detectors operated in real‐time.^[^ ^13^ ^]^

To assess the stability and the reliability of the detector's response we performed 10 repeated irradiation cycles. The signal variation resulted within 12% with respect to the mean value over 10 repetitions, as reported in Figure 3E. Due to the intrinsic fluctuations of the proton flux over different irradiation cycles the data points have been normalized by the relative impinging flux in order to be compared, and then by the mean value. Figure S12 (Supporting Information) reports the values of the currents without the normalization by the mean value.

The radiation hardness of the detector was also evaluated by measuring its response in pristine condition and after 40 min irradiation tests, corresponding to a total of 1.7 × 10^12^ protons impinging on the beam spot area. Figure 3F reports the variation of the signal amplitude relative to its pristine value. Few minutes after the test irradiations the measurement was repeated at the same conditions. A maximum variation of 14% was recorded, highlighting the radiation tolerance of the detector and further assessing its reliability as proton beam dosimeter.

## 3 Conclusion

In this work we report on the use of novel mixed 3D–2D perovskites films deposited from solution as active layers in flexible proton detectors. We deposited mixed 3D–2D perovskites films based on MAPbBr_3_ (3D) and (PEA)_2_PbBr_4_ (2D) and by tuning the 2D:3D ratio with a 35% of 2D precursor component we were able to combine the best features of both materials, targeting their use as direct radiation detectors. The presence of 2D perovskite allows to significantly lower the dark current and to achieve micrometer thick layers homogeneously covering the detector's active area, a very challenging goal to achieve with a pure MAPbBr_3_ active layer. On the other hand, the 3D perovskite allows to maintain a good sensitivity value, quite low in pure 2D perovskites due to its low density (2.27 g cm^−3^).

The response to 5 MeV protons has been assessed in a wide flux range [10^5^ – 10^9^] H^+^ s^−1^ cm^−2^ and under repeated sequential radiation exposures, up to 40 min of irradiation tests, corresponding to a total of 1.7 × 10^12^ protons impinging on the beam spot area. A maximum variation of 14% was recorded in the output signal, assessing the high radiation tolerance of the detector and its reliability as proton beam dosimeter. The minimum detectable dose rate resulted (72±2) µGy s^−1^, a value 2 orders of magnitude lower than the one recently reported in the only paper dealing with real‐time direct proton detectors based on organic thin films.^[^ ^13^ ^]^

The presented results on solution deposited 3D–2D perovskite films successfully address the open quest for novel functional materials able to directly convert ionizing radiation into an electrical signal and offer a seed challenge to further explore and boost research on material platforms targeting low‐cost scalability over large areas and mechanical flexibility.

## 4 Experimental Section

### Perovskite Synthesis and Device Fabrication

For the realization of the 3D/2D perovskite film, a solution was employed composed of the two precursor solutions mixed together. The first was a 3D perovskite (MAPbBr_3_) 1 m solution obtained by mixing PbBr_2_ (Sigma‐Aldrich >98%) and MABr (Sigma‐Aldrich >98%) in 1:1 molar ratio. The second solution was obtained by mixing PEABr (Sigma‐Aldrich >98%) and PbBr_2_ (Sigma‐Aldrich >98%) in 1:2 molar ratio to obtain a (PEA)_2_PbBr_4_ 1 m solution. For both solutions, the precursor powders were dissolved in *N*,*N*‐dimethylformamide (DMF) anidro and mixed overnight. The mixture to be deposited was prepared by adding 0.35 mL of 1 m PEA_2_PbBr_4_ solution to 1 mL of 1 m MAPbBr_3_ solution. The resulting solution was deposited by spin coating at 2000 rpm for 30 s on 125 µm thick PET substrates with prepatterned interdigitated gold electrodes. The metal electrodes were fabricated on plastic substrates by lithographic techniques. Before electrodes’ deposition the substrates were cleaned by subsequent ultrasonic baths in H_2_O and soap, deionized H_2_O and isopropyl alcohol. A n‐hexane and polydimethylsiloxane (PDMS) at 10:1 weight ratio is deposited on clean glass substrate to create an adhesive film. PET substrate is then laid down on the PDMS and heated at 100 °C for 10 min on a hot plate. A positive photoresist (S1818) is then spin coated on the PET surface at 4000 rpm for 60 s. The layout has been projected exposing the resist through an optical Microwriter (ML3 Durham Magneto Optic). The resist has been developed by MF‐139 and then rinsed with deionized water. Gold/chromium electrodes were thus deposited through thermal vacuum evaporation and patterned by dipping the whole structure into acetone bath for 4 h. The resulting interdigitated electrodes have 30 µm channel length, 9.6 mm channel width and 1 mm^2^ total area.

### GI‐XRD Measurements

GIXRD measurements were performed at the X‐ray Diffraction beamline 5.2 at the Synchrotron Radiation Facility Elettra in Trieste (Italy). The X‐ray beam emitted by the wiggler source on the Elettra 2 GeV electron storage ring was monochromatized by a Si(111) double crystal monochromator, focused on the sample and collimated by a double set of slits giving a spot size of 0.2 × 0.2 mm. The beam was monochromatized at 1.0 Å. Measurements were performed at a temperature of 293 K.

Samples were oriented by means of a kappa diffractometer with a motorized goniometric head. To explore the reciprocal space as much as possible the alignment allowed to spin the sample around an axis perpendicular to its surface, with the possibility to vary the X‐ray impinging angle.

Bidimensional diffraction patterns were recorded with a 2 m Pilatus silicon pixel X‐ray detector (DECTRIS Ltd., Baden, Switzerland) positioned perpendicularly to the incident beam, at a distance of 150 mm from the sample.

Incidence angles were kept close or slightly over 0.05°, the critical angle at the chosen wavelength. Uncertainty in the precise assessment of the incidence angle is estimated to be around 0.02°.

Patterns were calibrated by means of a LaB_6_ powder standard from NIST by means of the software GIDVis^[^ ^31^ ^]^, which also allowed to represent the diffracted intensity as a function of reciprocal lattice vectors components q_xy_ and q_z_ and to project on the image predicted positions for the different phases present in the samples.

Predicted positions were drawn using cif files or cell information from the following sources:

MAPbBr_3_ perovskite phase, (COD: 1545320):^[^ ^32^ ^]^
Space group Pm‐3m (221) – cubic
*a* = 5.9195 Å; *b* = 5.9195 Å; *c* = 5.9195 Å
*α* = 90°; *β* = 90°; *γ* = 90°
Orientation (0 0 1)
(PEA)PbBr_4_ phase (COD: 2 224 096)^24^:
Space‐group P‐1 (2) – triclinic
*a* = 11.6150 Å; *b* = 11.6275 Å; *c* = 17.5751 Å
*α* = 99.5472°; *β* = 105.7245°; *γ* = 89.9770°;
Orientation (0 0 1)
PET phase:^[^ ^33^ ^]^
Space‐group P‐1 (2) triclinic
*a* = 4.56Å, *b* = 5.94 Å, *c* = 10.75 Å,
*α* = 98.5°, *β* = 118°, *γ* = 112°;
Orientation (1 0 0)

It appears that the MAPbBr_3_ perovskite phase is well‐oriented both in the substrate plane and perpendicularly to it, while (PEA)_2_PbBr_4_ shows clear spots only in the out of plane direction, denoting a multi‐layered organization with repetition parameter c, showing poor long‐range order in the substrate plane.

### AFM Measurements

AFM measurements are performed using a Park NX10 system using PPP‐NCHR tips (Nanosensors) in noncontact mode and applying adaptive scan‐rate to slow down scan speed at crystallite borders. AFM images were processed through Gwyddion software.

### Transient Response Measurements

Excitation source used for time response measurement was a PicoQuant picosecond laser diode (375 nm, 40 ps pulse duration, power of 5.3 mW) with Taiko PDL M1 driver. Using built‐in bunch mode, 50 ms laser burst with 50% duty cycle was obtained. The light‐induced current signal was converted to voltage using a current amplifier (FEMTO DHPCA‐100, Gain 10^7^ V A^−1^, bandwidth 1.8 MHz), then the signal was acquired with a Rohde & Schwarz RTB 2004 oscilloscope at 500 kHz. During the measurement the sample was biased at 10 V through the current amplifier.

### Photoluminescence Measurements

Photoluminescence spectra were obtained by means of the same laser diode employed in transient response measurements, at a power of 2.98 mW in continuous mode. The PL emission on the sample surface is collected and guided with an optical fiber into a CCD compact spectrometer (ThorLabs CCS200). The optical fiber is positioned at 45° with respect to the incident laser beam and a 400 nm high pass filter is positioned before the optical fiber to cut the excitation source.

### Photocurrent Measurements

During the PC measurements, the sample was biased at 9 V. These measurements were carried out in air and at room temperature using a 150 W Xenon arc lamp, which has a broad emission spectrum for *λ* ≥ 250 nm. The light was chopped mechanically at a low frequency (<120 Hz) and filtered by a grating monochromator. A long‐pass wavelength filter has been used for spectrum above 410 nm. The photogenerated current was acquired with a SRS830 lock‐in amplifier locked to the chopper frequency.

### Proton Irradiation and Detector Characterization

The mixed 3D–2D perovskite film‐based detectors were characterized using a 5 MeV proton beam provided by the 3 MV Tandetron accelerator of the LABEC ion beam center (INFN Firenze, Italy). The proton beam is extracted into ambient pressure through a 200 nm thick Si_3_N_4_ membrane; the sample has been installed 8 mm far from the extraction window. Proton beam currents used in this work are typically in the 0.1–100 pA range. The weak intensity of the extracted beam is monitored and quantitatively measured using a rotating chopper, placed between the silicon nitride window and the sample; the chopper is a graphite vane covered with a thin nickel evaporation, and the Ni X‐ray yield is used as an indirect measurement of the beam current. To determine the actual energy of the protons impinging onto the 4 µm thick perovskite layer, the energy lost by the protons passing through the several layers interposed between the beam and the sensor, namely, 200 nm of Si_3_N_4_ for the beam extraction window, 8 mm of mixed air‐He (50–50%) atmosphere in the gap between the extraction window and the metal box, 14 µm of Al for the entrance window of the box, where the sensor was enclosed, and 14 mm of air inside the box, has to be calculated. After passing through these layers, protons lose about 390 keV, as calculated with the SRIM Monte Carlo code.^[^ ^27^ ^]^

During the tests samples were enclosed in a small Faraday cage to lower the electromagnetic noise and for keeping the samples in dark. Samples were irradiated with proton flux in the range [10^5^ –10^9^] H^+^ cm^−2^ s^−1^ and 10 s time window. The spot of the proton beam has an area of 0.17 cm^2^.

Electrical response under proton beam irradiation was acquired by a Keithley 2614B precision Source/Measure Unit.

## Conflict of Interest

The authors declare no conflict of interest.

## Supporting information

Supporting Information

## Data Availability

The data that support the findings of this study are available from the corresponding author upon reasonable request.
