# Supplementary material for: Mixed 3D–2D Perovskite Flexible Films for the Direct Detection of 5 MeV Protons
Source: Adv Sci (Weinh). 2022 Nov 27;10(1):2204815. doi: 10.1002/advs.202204815 (PMC9811469; doi:10.1002/advs.202204815)
Supplement: Supplementary file 1 — Supporting Information [file ADVS-10-2204815-s001.pdf]

ADVANCED SCIENCE
Open Access

Supporting Information

for *Adv. Sci.*, DOI 10.1002/advs.202204815

Mixed 3D–2D Perovskite Flexible Films for the Direct Detection of 5 MeV Protons

Laura Basiricò, Ilaria Fratelli, Matteo Verdi, Andrea Ciavatti, Luisa Barba, Olivia Cesarini, Giorgio Bais, Maurizio Polentarutti, Massimo Chiari and Beatrice Fraboni*

Supporting Information

Mixed 3D-2D perovskite flexible films for the direct detection of 5 MeV protons

Laura Basiricò, Ilaria Fratelli, Matteo Verdi, Andrea Ciavatti, Luisa Barba, Olivia Cesarini, Giorgio Bais, Maurizio Polentarutti, Massimo Chiari, Beatrice Fraboni*

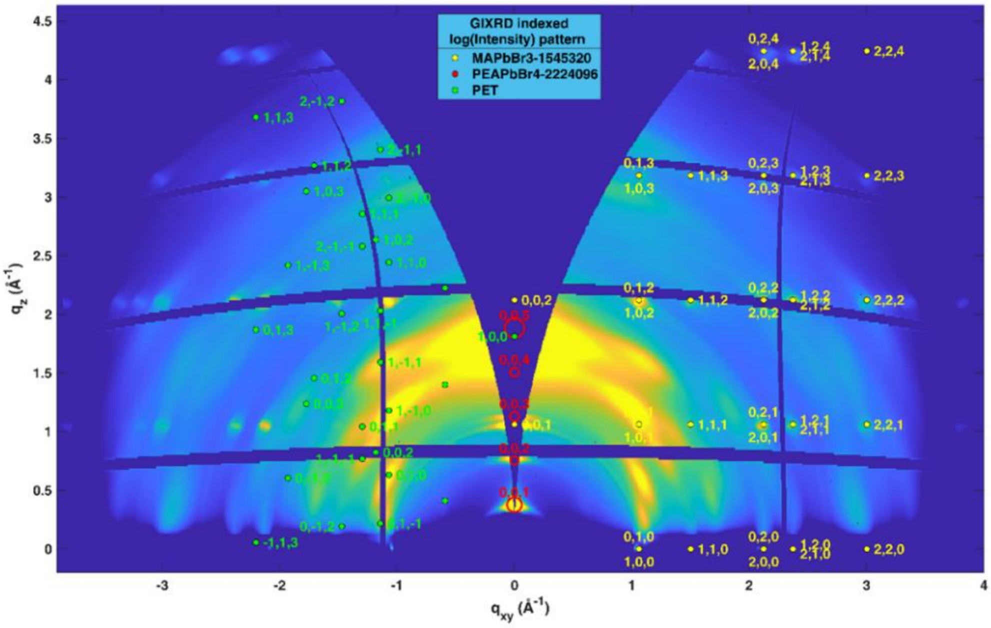

Fig. S1: X-ray diffraction pattern in grazing incidence geometry of mixed 3D-2D perovskite film, at 2D:3D 35% precursors volume ratio, representing square root of diffracted intensity as a function of reciprocal lattice vector components q_{xy} and q_z. On the image are reported expected diffraction spots positions and indexes from the three components of the sample, namely crystalline $MAPbBr_3$ (yellow) and multilayered $(PEA)_2PbBr_4$ (red), both oriented with **c** axis perpendicular to the sample surface, and PET from sample substrate, with **a** axis out of sample surface.

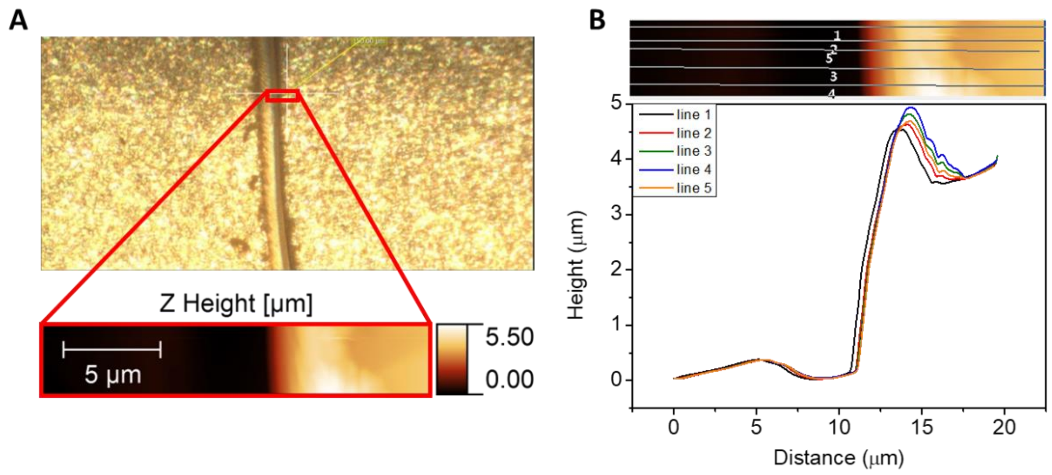

Fig. S2: AFM thickness profile of the mixed 3D-2D perovskite active layer. A) The thickness of the mixed 3D-2D perovskite active layer was assessed by making a scratch by means of a scalpel along the material film (optical image on the top) and acquiring an image of the area involved (5 µm x 3 µm) (bottom). B) The final thickness is calculated as the average value of five different profiles extracted along the entire length of the groove.

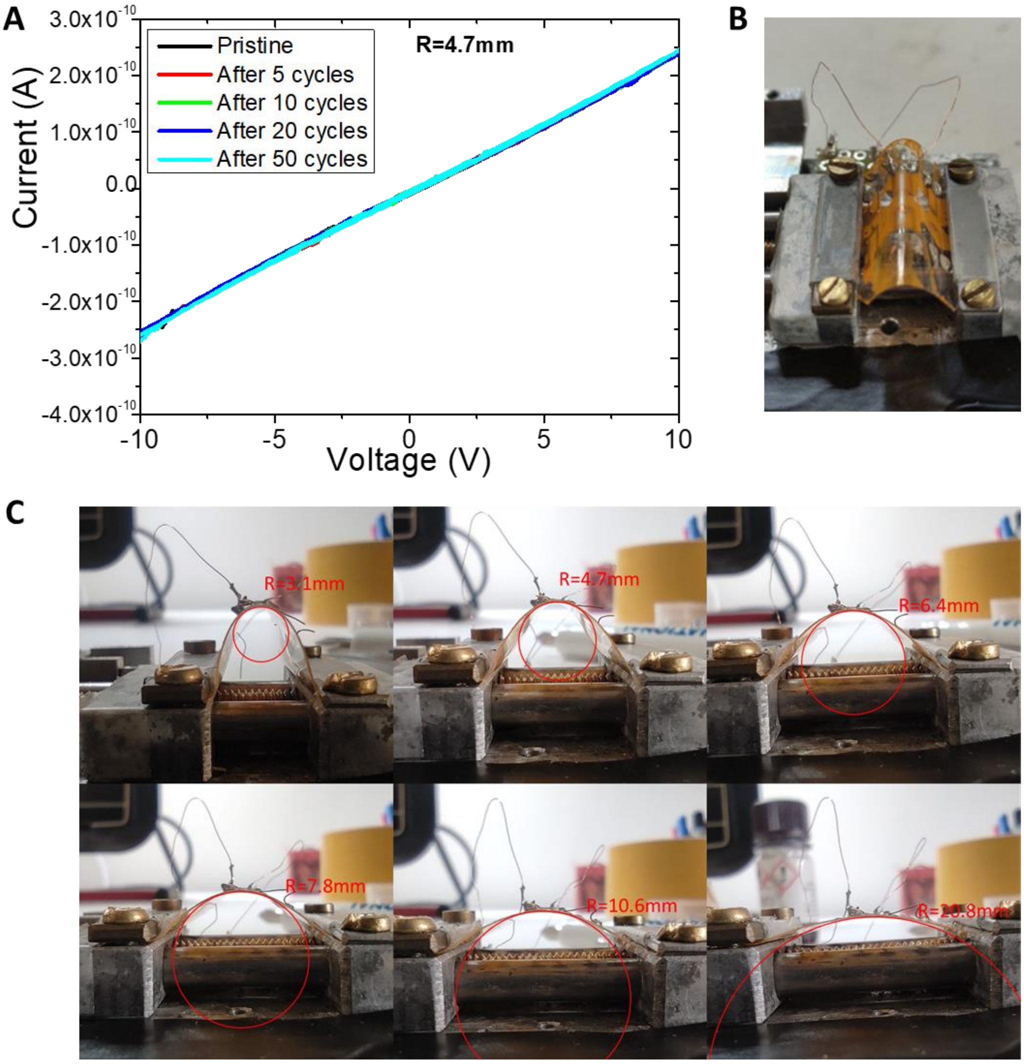

Fig. S3: Bending test. A) Current vs. Voltage measurement of the device in pristine flat condition and after multiple bending cycles (up to 50) at 4.7 mm bending radius. B) Detail of the experimental set-up used for the characterization of the detector during bending. C) Picture showing the estimation of bending radius of the bending test.

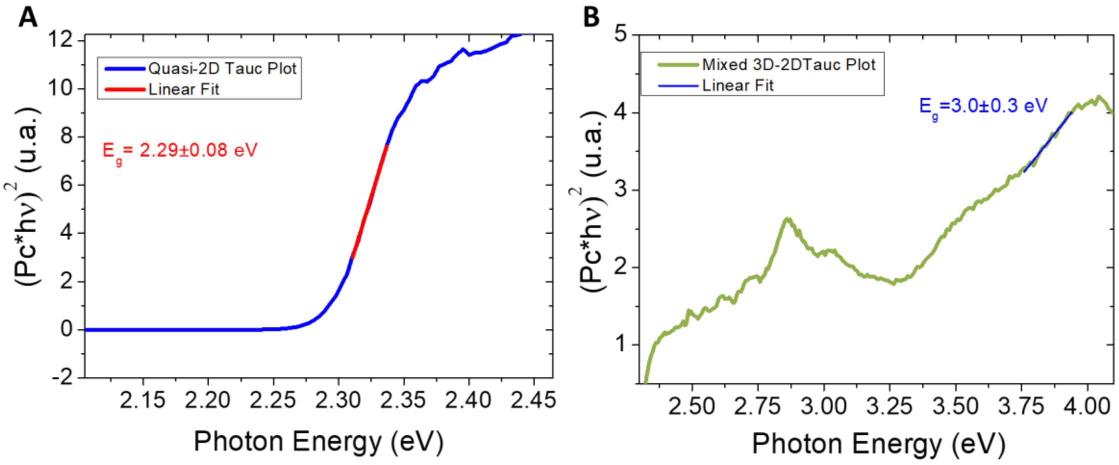

Fig. S4: Tauc plot from the UV-visible photocurrent spectrum of the mixed 3D-2D active layer. Experimental data and fit lines for the estimation of the energy gap according to Tauc's method of $MAPbBr_3$ (A) and $(PEA)_2PbBr_4$ (B).[34]

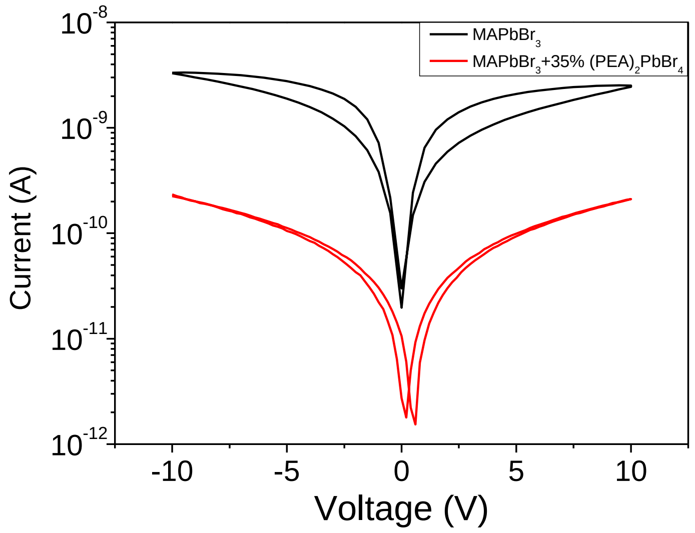

Fig. S5: Current versus voltage characteristics of 3D $MAPbBr_3$ (black line) and mixed 3D-2D

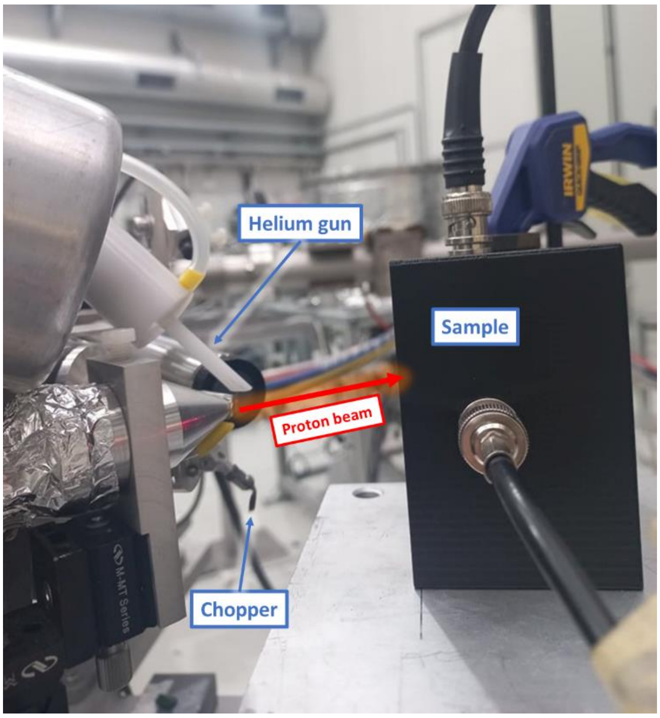

Fig S6: 5 MeV proton irradiation at the LABEC ion beam laboratory using an extracted beam line and tuning the flux of protons in the range [10^5 - 10^9] H^+ cm^{-2} s^{-1}.

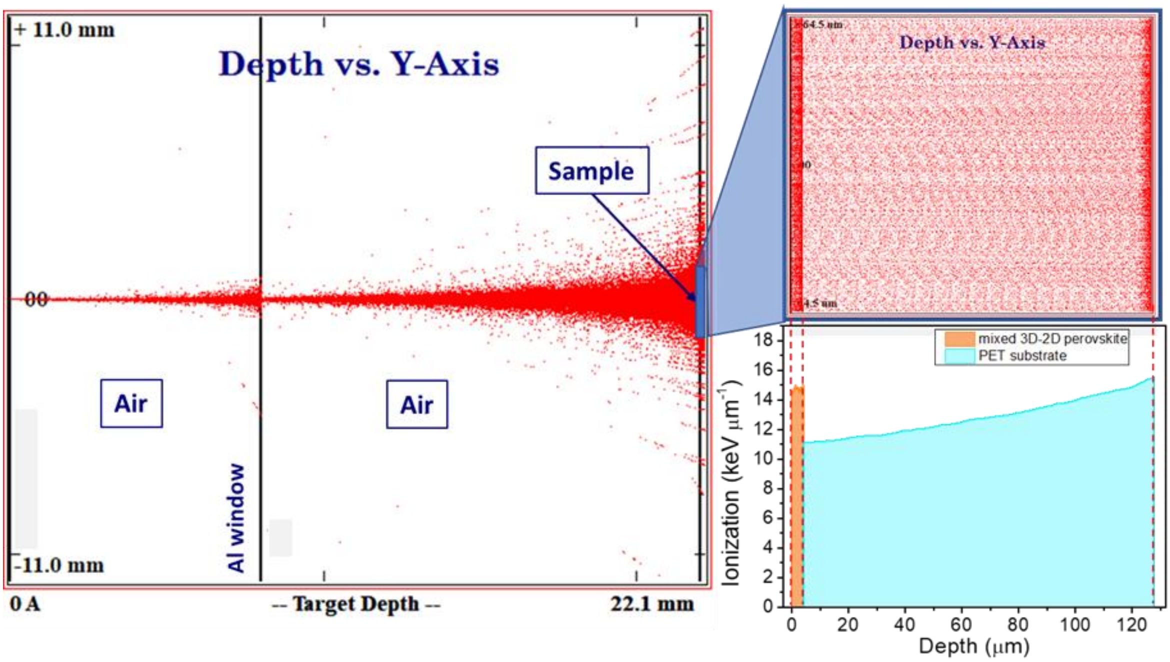

Fig. S7: 5 MeV proton penetration from beam source to sample (left) and within the perovskite and substrate layers (right).

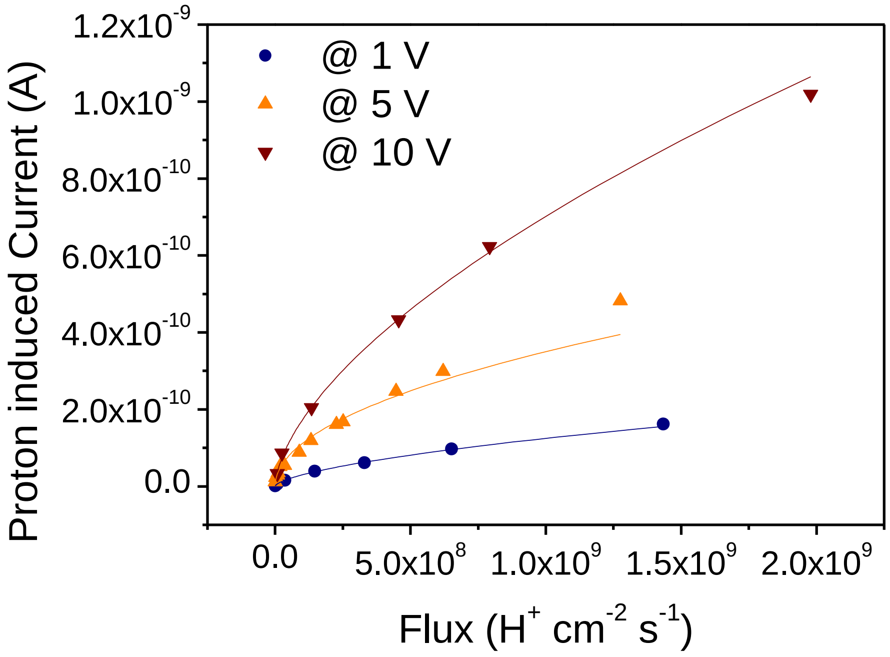

Fig S8: Plot of the proton induced current in function of the proton flux in linear scale. During the measurement proton irradiation time window was set at 10 s and the samples were biased at 1 V (blue circles), 5 V (orange triangles) and 10 V (upside down red triangles).

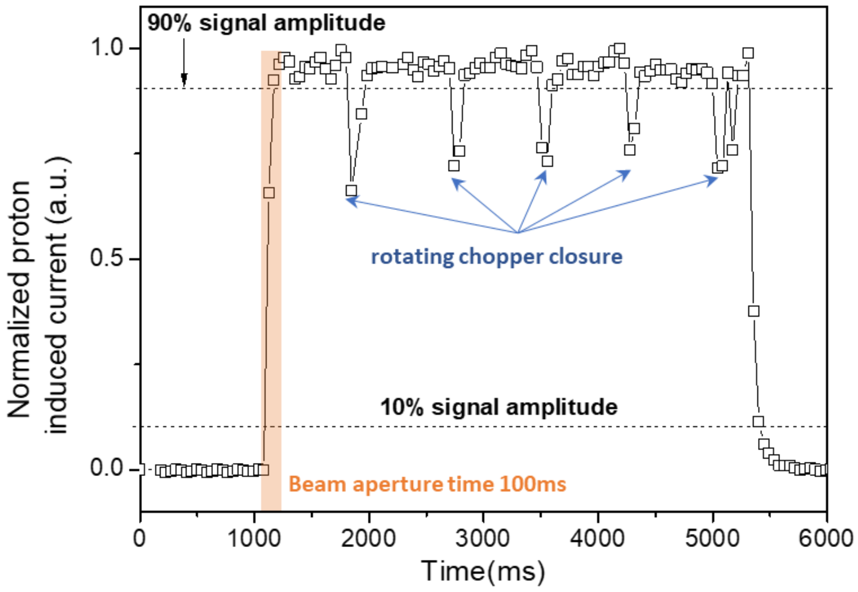

Fig. S9: Timing of the signal under proton beam. Plot of the proton induced current normalized for its maximum value in function of time. The orange shadow shows the beam aperture time window at LABEC. The arrows indicate the rotating chopper transit in front of the sample.

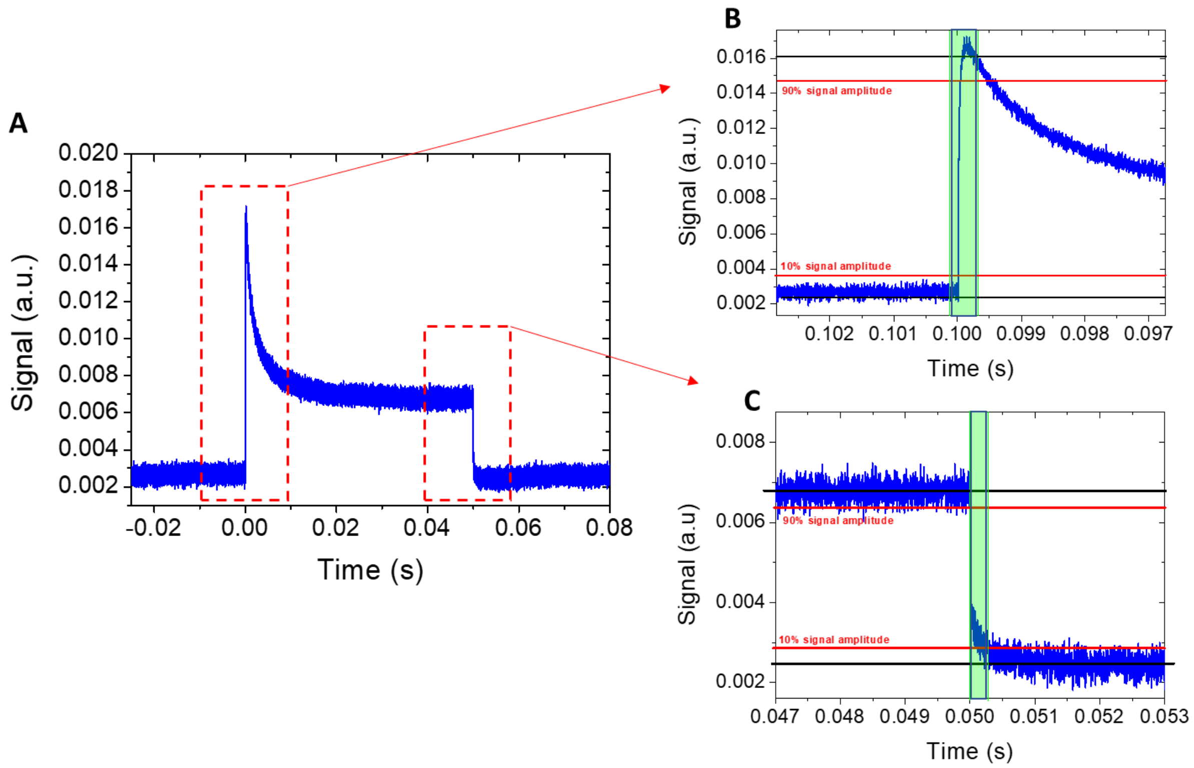

Fig. S10: Evaluation of rise and fall time under laser diode irradiation. A) Whole signal recorded, the two dashed boxes indicate the zoomed plot of the signal rise and the signal fall, reported in B) and C) respectively.

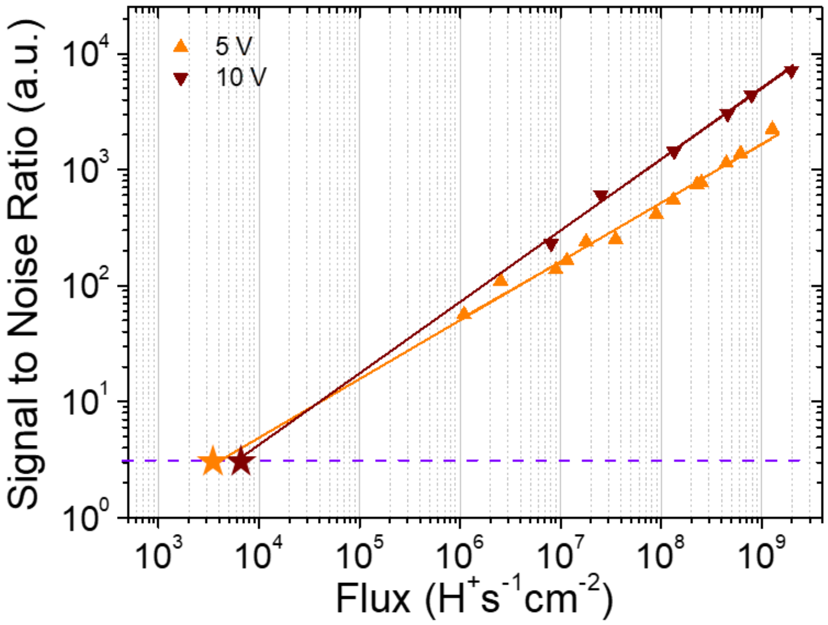

Fig. S11: Determination of LoD at 5 V and 10 V bias voltage. Plot of the SNR in function of the proton flux. The red star indicates the LoD value extrapolated from data at 10 V, while the orange star that from data at 5 V.

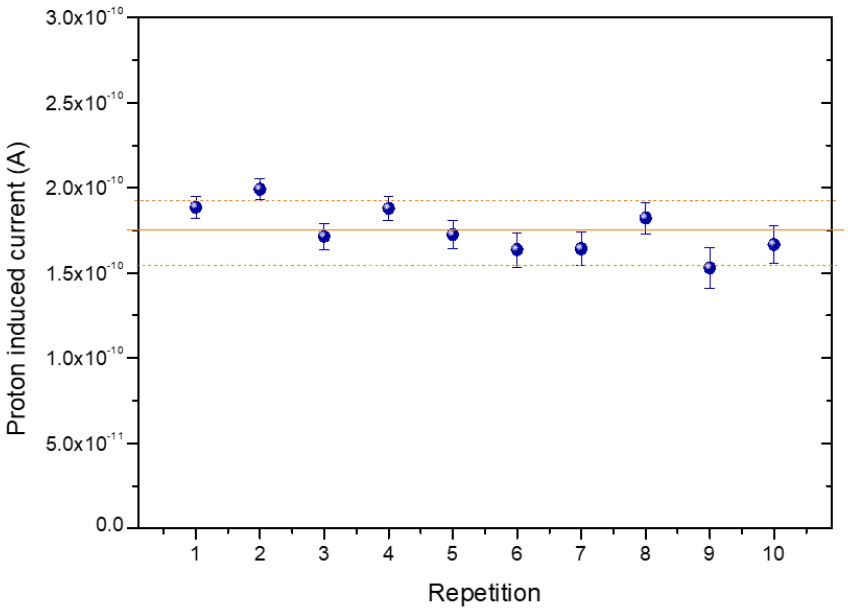

Fig. S12: Stability of the proton induced current variation up to 10 repetitions of 10 s irradiation cycles. Each data point has been normalized for the impinging proton flux. The uncertainty associated to each point has been evaluated from the root sum square of the statistic uncertainty of the proton-induced current value and that associated to the proton flux measurement. The dashed orange lines identify the maximum semi-dispersion around the mean value, i.e. half of the difference between the maximum and the minimum value of the data set.
